# VisNet: Deep Convolutional Neural Networks for Forecasting Atmospheric Visibility

**DOI:** 10.3390/s19061343

**Published:** 2019-03-18

**Authors:** Akmaljon Palvanov, Young Im Cho

**Affiliations:** Department of Computer Engineering, Gachon University, Gyeonggi-do 461-701, Korea; akmaljon.palvanov@gmail.com

**Keywords:** convolutional neural networks, Fast Fourier transform, spectral filter, visibility, VisNet

## Abstract

Visibility is a complex phenomenon inspired by emissions and air pollutants or by factors, including sunlight, humidity, temperature, and time, which decrease the clarity of what is visible through the atmosphere. This paper provides a detailed overview of the state-of-the-art contributions in relation to visibility estimation under various foggy weather conditions. We propose VisNet, which is a new approach based on deep integrated convolutional neural networks for the estimation of visibility distances from camera imagery. The implemented network uses three streams of deep integrated convolutional neural networks, which are connected in parallel. In addition, we have collected the largest dataset with three million outdoor images and exact visibility values for this study. To evaluate the model’s performance fairly and objectively, the model is trained on three image datasets with different visibility ranges, each with a different number of classes. Moreover, our proposed model, VisNet, evaluated under dissimilar fog density scenarios, uses a diverse set of images. Prior to feeding the network, each input image is filtered in the frequency domain to remove low-level features, and a spectral filter is applied to each input for the extraction of low-contrast regions. Compared to the previous methods, our approach achieves the highest performance in terms of classification based on three different datasets. Furthermore, our VisNet considerably outperforms not only the classical methods, but also state-of-the-art models of visibility estimation.

## 1. Introduction

The transparency of the atmosphere is a meteorological variable, and is known as the meteorological optical range or atmospheric visibility in the Glossary of Meteorology. According to the World Meteorological Organization [1], International Commission on Illumination (CIE) [2], and American Meteorological Society [3], visibility is defined as the longest distance at which a black object of suitable dimensions, located on or near the ground, can be seen and readily identified when observed against the horizon sky. When the observer is a camera, the visibility is the maximum visible distance from the camera.

Visibility estimation is often viewed as one of the most important services provided by the meteorological profession [4]. Since fog is a collection of water droplets or water-saturated fine particles, or even fine ice crystals exhibiting the hygroscopic characteristic, it significantly decreases the horizontal visibility of a scene [5,6]. Low-visibility conditions, due to dense fog, mist, or their combination, present numerous challenges to drivers and increase the risk for passengers, including strong visual effects, decreasing the ability to see through the air. These challenges arise due to the reduction in the luminance contrast. The scattered light results in a complete loss of object detection, making object recognition tough and considerably increasing the visual response time. This, in turn, reduces the operational capacity of vehicles and leads to fatal accidents on roads (chain collision). In fact, according to the statistics provided by National Highway Traffic Safety Administration of the US [7] and the Foundation for Traffic Safety [8], 28% of all crashes occur under inconvenient weather conditions, and according to the World Health Organization [9], one person dies every 25 s because of road injuries. Hence, under perturbed atmospheric conditions, the estimated average of total annual accidents is around 31,385, road fatalities are over 511, and each year, almost 12,000 injuries occur due to accidents in low-visibility climates [9,10]. Concurrently, low visibility is directly related to the aviation industry as it can trigger ample airport delays and cancellations [11,12]. This not only brings enormous losses for airports and airlines, but also affects public travels. Furthermore, it is a common cause of flight accidents [4,13,14,15,16]. Similar to flight safety, low visibility also affects water transport operations in terms of navigation, orientation, etc. [17,18]. Therefore, an accurate estimation of visibility is a major parameter to maintain safety.

On the other hand, visibility reduction causes difficulty for not only human observers, but also computer vision algorithms [19]. Degradation of visibility strongly decreases the image or video quality of captured outdoor scenes. Visibility is much better under clear weather conditions than that in air polluted with considerable water droplets or dust particles from the atmosphere. The key reason for the degradation of image quality under foggy or misty conditions is considerable suspended fog or mist particles in the air, which leads to the diffusion of most light prior to it reaching the camera or other optical devices. As a repercussion, the whole image gets blurred [20].

According to the British Meteorological Service [21], the scale of visibility can be classified into 10 classes, ranging from “zero” to “nine” (from dense fog to excellent visibility). Dense fog appears when the prominent objects are not visible at 50 m, while excellent visibility occurs when prominent objects are visible beyond 30 km. Low-visibility is a normal atmospheric phenomenon, but predicting it accurately is extremely complicated notwithstanding that weather forecasting techniques have improved over the years. There are two major approaches for measuring visibility: Sensor-based and visual performance-based. Visibility can be measured using weather sensors, forward scatter visibility sensors, transmissometers, and nephelometers. However, the network of meteorological measurement is not dense enough to be able to detect all occurrences of fog and mist [12,22]. In the case of visibility meters, there are two fundamental problems to convert atmospheric parameters into visibility. First, visibility is a complex multivariable function of many atmospheric parameters, such as light scatter, air light, light absorption, and available objects, and measuring every possible atmospheric parameter to derive human-perceived visibility is simply too complex and costly [23]. The second is expressing the spatially variant nature of atmospheric visibility using a single representative value, i.e., distance. This can work only if the atmosphere is uniform, which, however, occurs rarely [23]. The nephelometers use a collocated transmitter and receiver to measure the backscatter of light off particles in the air. However, they are sometimes error-prone as they miss many of the essential phenomena affecting how far an observer can truly see [24,25]. Beyond this, there are other drawbacks in that most of this equipment is very expensive and needs high installation requirements [12].

An alternative method for measuring visibility is visual performance, which leverages pre-installed surveillance or closed-circuit television (CCTV) camera systems, and, therefore, is much more feasible. Forecasting atmospheric visibility via a camera might have superiorities in both financial and efficiency aspects, as, at present, numerous safety and traffic monitoring cameras are deployed globally and various vision-based applications are in wide use. In detail, the United States acquires approximately 30 million surveillance cameras and 4 billion hours of footage per week [26]. Moreover, in the United Kingdom and South Korea, the estimated total number of installed CCTV cameras is 5 million and 1 million, respectively [27,28]. Most of these camera videos and images are available on the Internet and can be used for almost real-time access. However, the deployment of cameras increases both the amount and complexness of camera imagery, and thus, automated algorithms need to be developed to be simply used operationally by users [29]. Although camera-based visibility estimation is closer to visual performance, most of the present studies still rely on statistical analysis of collected meteorological data [11,14,16,30] and require additional hardware [24,31] or further efforts [23,32,33]. Other existing approaches that have revealed satisfactory results are model-driven and are not verified by big data forgathered from real-world conditions [18,34] or are static based on a single image [35,36]. The most critical issue regarding these techniques is estimation capability. As most of the optics-based methods can estimate only short-visibility distances within 1–2 km [16,37,38,39,40], deploying them in the real world to evaluate longer visibility distance is tough.

Even if the visibility is uniform in all directions, accurate estimation is still a complex problem. Therefore, to tackle the limitations of existing software, as well as the drawbacks of optics-based visibility estimation methods, we propose a vision-based visibility estimation model by employing state-of-the-art convolutional neural networks (CNNs). With the recent improvements in computational tools and computer performance, CNNs have achieved significant advancements in different tasks. They have demonstrated high performance on classification, segmentation, understanding image content, and detection [41,42,43,44,45,46], and showed great success in evaluating remote sensing imagery [47,48]. Except for the recent advancements in CNNs, their several characteristic features have inspired us to employ them in the field of meteorological forecasting. In addition, they have the ability of solving hetero-associative problems [49]. To represent the weather situations, extracting various weather characteristics is too complex, but CNNs can automatically extract effective features from the data. CNN-based models are capable of representing a phenomenological approach to predict the features of complex systems [50], the atmosphere in this case. The other key factors behind successful visibility estimation are large labeled datasets that support the learning process and novel network architectures with hundreds of millions of parameters [51].

Our data-driven approach capitalizes on a large collection of real-world images to learn rich scenes and visibility varieties. From the practicality standpoint, there is no publicly available large database of annotated images from real-world conditions for visibility estimation and fog detection, especially in the case of long-range visibility. Thus, we collected a largest dataset for this problem, which consists of more than 3 million CCTV camera images with the exact visibility values. We term the collected dataset in this paper as Foggy Outdoor Visibility Images (FOVI). Most of the existing methods have large ranges between classes; only a few proposed approaches have focused on long-range visibility estimation, while most methods have focused on short ranges. Thus, we performed extensive experiments on the FOVI dataset for two visibility ranges: Long-range (from 0 to 20 km) and short-range (within 0 to 1 km). To evaluate our model’s effectiveness objectively, we evaluated the performance of the current state-of-the-art models on the FOVI dataset. Additionally, to reveal the effectiveness of the proposed model fairly, we conducted experiments on the currently known Foggy ROad Sign Images (FROSI) dataset [52], which is a small-sized existing dataset of synthetic images. We analyzed the performance of the model more deeply for short-range (from 0 to more than 250 m) on the FROSI dataset, and compared the results with those of the previously proposed approaches.

We proposed a system that is specially designed to estimate visibility from camera imagery. Our focus was on developing a model that can adapt to different visibility ranges. The VisNet is a robust model that receives outdoor images (RGB) captured during the daytime and processes each image prior to feeding them into a classifier that then gives the result, which is the visibility distance, according to given inputs. Generally, it can be compared with any CNN based classification model, but the main difference is having a pre-processing step. The proposed model comprises two phases: Data pre-processing and classification. First, all input images are processed so that the classifier can learn different hidden features of the inputs in the next phase. For classification, we developed a deep integrated CNN architecture, which learns triple inputs simultaneously using a small-size convolutional kernel. During pre-processing, (1) the input image is filtered in the frequency domain using the fast Fourier transform (FFT) algorithm and a high-pass filter and (2) the same input image is applied to a spectral filter. In the first type of image, the model focuses on mostly local features, looks for only visible objects, and ignores fog, and cloud and low-frequency features are removed during filtering. In contrast, the second type of image illustrates fog or low-contrast features. The deep integrated CNN was specially implemented for estimating visibility in dissimilar foggy weather conditions. Therefore, in this paper, we use the term of VisNet as the name of our proposed model. Extensive experiments showed that the VisNet achieves better performances than the current state-of-the-art models in all three visibility ranges. Our major contributions compared to the existing approaches can be summarized as follows:

First Contribution: In the metrological field, this is the first proposed application of a deep integrated CNN model that uses the triple stream of input images to tackle the problem of visibility estimation. The VisNet is robust, extracts features automatically, and does not need additional hardware and further efforts, such as manual feature extraction or pre-installed targets. Moreover, our model achieves better results in different ranges and two different datasets. We provide experimental results and benchmarks on previously used models, including the state-of-the-art comparison.

Second Contribution: We compiled a large-scale dataset of images based on real visibility scenes and visibility values of each image. The dataset includes more than 3 million images of real-world scenes, including nighttime views.

Third Contribution: We conducted complex experiments as the VisNet trained and tested on two absolutely dissimilar datasets for three different visibility ranges with three different classes (41 classes in long-range, 21 and 7 classes in short-range).

Fourth Contribution: We provide a promising way of removing low-frequency pixels from images (features, such as cloud, fog, and sea, which are essential for evaluating the visibility level of the visible feature in the image, using the proposed method). To the best of our knowledge, this is the first time that the FFT-based filtered images are used for visibility estimation. Outputs of this filter are trained simultaneously with spectral filtered and original images.

Fifth Contribution: To extract low-contrast regions from the image, we implemented a filter with a view to extract the fog effect. The filter specially implemented on a basis of a pseudocoloring and color map. The low contrast regions of an input image are revealed by applying this spectral filter, mostly foggy and low-cloud captured regions. These spectral filtered images are simultaneously trained with the FFT filtered images and original images.

This paper is structured as follows. Section 1 describes the research background and significance. Section 2 reviews related studies. Section 3 focuses on the methods and materials, including the overall structure of the VisNet model, pre-processing methods, and classification. Section 4 presents the experimental results and discussion, and finally, Section 5 presents the research summary.

## 2. Related Works

In this paper, we discuss research conducted on visibility estimation divided into two broad streams: Methods based on artificial neural networks (ANNs) and statistical approaches.

### 2.1. ANNs-Based Methods

Unlike traditional statistical techniques, ANNs have huge potential in applications to forecasting visibility, as they are well-suited to tackle complex interactions. ANNs approximate virtually any continuous nonlinear function with arbitrary accuracy, and thus, are known as the universal function approximators [53]. Although ANNs have existed for over 60 years, they were first used in atmospheric science in 1986 [54,55]. Subsequently, ANNs were applied to detect tornados in 1996 [56,57], and to forecast quantitative precipitation in 1999 [58]. At that time, multilayer perceptron (MLP) was the most commonly used ANN algorithm for atmospheric science [59].

In meteorology, the first attempt at improving a short-range visibility forecast using ANNs was presented by Pasini et al. [60]; this problem was not faced in previous ANN literature. They implemented a simple feed-forward neural network with a single hidden layer to learn metrological data. A similar study for mapping the complex and nonlinear relations between visibility and multiple atmospheric inputs was proposed in Ref. [61]. A new methodology of using ANN for fog forecasting was introduced in Ref. [17]. The authors implemented a neural network that received eight input variables in the initial layer and propagated to five neurons in the next layer. Finally, the output layer had a single neuron, which corresponded to the presence or absence of fog. Based on the principle that low visibility has higher risk, a new risk neural network model was implemented in Ref. [62], which outperformed the standard ANN and linear regression models [62].

Recently, Chaabani et al. [63] achieved great performance using ANNs, where the visibility distance under foggy weather situations was estimated using a camera. The model could estimate the short-range distance (60–250 m) with an overall accuracy of 90.2%, using a single hidden layer with 12 neurons and synthetic images used as input descriptor vectors [63]. However, real-world data are too complex to handle with similar ANN-based models. In this case, only a few CNN-based data-driven methods were proposed. Li et al. [12] proposed pre-trained AlexNet [64] to estimate the visibility distance from webcam images by taking advantage of CNN features. The model could evaluate up to 35 km with a 77.9% training and 61.8% testing accuracy. Similarly, in Ref. [29], another data-driven approach was introduced to estimate the relative atmospheric visibility using outdoor images collected from the Internet; the model was named CNN-RNN because it combined CNNs and recurrent neural networks (RNNs) [29]. Shortcut connections bridged them for separate learning of the global and local variables of inputs. Eventually, CNN-RNN [29] could achieve a 90.3% accuracy, which is a quite promising result for this task. The evaluation capacity of the model was 300–800 m. In the case of multi-label classification tasks, Kipfer and Kevin [37] conducted experiments on very deep, well-known models, namely ResNet-50 [65] and VGG-16 [66], as well as baseline models, such as logistic regression [67] and SVM (Support Vector Machine) [68]. ResNet-50 is a version of residual networks [37] comprising 50 layers, aiming to build ever larger, ever deeper networks for solving more complex tasks. The network learns residuals by simply adding a shortcut connection that directly connects the input of a layer to its output [65,69,70,71]. VGG-16 [66] is a version of very deep CNNs for large-scale image recognition, and was the winner of the ImageNet Challenge 2014 in localization and classification tracks [72].

Visibility reduction because of the fog phenomenon has been broadly evaluated through the application of ANN models. An assessment of the ability of ANNs to forecast fog was studied in Refs. [4,13,14], and [73]; however, these methods used further meteorological data (wind direction and speed, horizontal visibility, humidity and pressure, air temperature, etc.) to achieve accurate results. Another model that relied on similar meteorological data was given in Ref. [16], where a deep ANN model was implemented in airport visibility forecast. However, deploying the abovementioned models to solve the real-world problem is costly as gathering such data requires even further use of hardware.

### 2.2. Statistical Approaches

The first implementation of sensing weather situations using camera imagery was performed for the USA Army for the support of their ground operations on the battlefield [74]. The authors utilized digital cameras deployed for military services to monitor enemy forces, and to gather and analyze real-time weather data [75]. Later studies [25,76,77,78] developed an algorithm for visibility monitoring using digital image analysis.

The recent approach proposed in Ref. [15] used the probabilistic nowcasting method for low-visibility conditions in cold seasons, where standard meteorological measurements were used as inputs and slight improvements among similar approaches were obtained. A tree-based statistical method and piecewise stationary time-series analysis were proposed in Refs. [11,36]. Fog detection and visibility estimation of the road scene by extracting the region of interest were studied in Refs. [32,79]. An approach that needs pre-installed targets located at different distances was presented in [80]. Based on the contrast sensitivity function of the human visual system, Tarel et al. [80] implemented a computer vision algorithm by applying Koschmieder’s law [36] to none-dark objects to evaluate visibility. Comparisons of images computationally evaluated with an image sensor and optically estimated with a visibility sensor were also detailed in Ref. [80]. Viola et al. investigated how the characteristics of vehicle rear lights and fog affect distance perception [39]. A new stereovision-based technique and analyses of different optical sensors for measuring low visibility were given in Ref. [38].

The use of a charge-coupled device (CCD) video camera for measuring the brightness contrast of a black object, against its background and estimated visibility, was presented in Ref. [23]. The development of an algorithm for visibility monitoring using digital image analysis was studied in Refs. [25,76,77,78]. Unlike data-driven approaches, [18] presented a model-driven approach for monitoring the meteorological visibility distance through ordinary outdoor cameras.

Several edge detection algorithms have been widely used to predict visibility distance from an image. The Canny edge detection and region growing algorithm were proposed in Ref. [40], and a combination of the average Sobel gradient, dark channel prior (DCP), and region of interest were introduced in Ref. [81]. The comprehensive visibility indicator [82] has achieved great success as compared to DCP [83] and entropy [84] methods in the challenge of visibility forecasting. The landmark discrimination technique using edge detection, contrast attenuation, contrast reduction between couple of targets, and global image features was studied in detail in Ref. [85]. In the context of the automated vehicle and advanced driver assistance system, visibility estimation has been extensively studied for both daytime [86,87] and nighttime [88]. Clement et al. [89] developed a generic sensor of visibility using an onboard camera in a vehicle. The method proposed in [90] explained how to gain a spatial partial structure reconstruction to estimate the visibility distance using the estimation of vehicle motion, as well as images obtained from the onboard camera filming the scene. The system of forward looking vision, which can simultaneously track the lane and estimate visibility, was proposed in Ref. [24]; visibility estimation was performed by measuring the contrast attenuation between consistent road features ahead of the moving vehicle. A target-based static model for moving vehicles was proposed in Refs. [31,35].

This problem was tackled from different perspectives. For instance, fog detection has been extensively studied [91]. To test the reaction of drivers, the authors in Ref. [92] conducted a very interesting experiment called “FOG,” where a prototype of a small-scale fog chamber, which can produce stable visibility levels and homogeneous fog, was developed. The DCP method is commonly applied for atmospheric visibility [82], image dehazing [93,94], and image defogging [95,96]. Table 1 summarizes the comparison results of previous studies.

## 3. Methods

### 3.1. Model

Although deep CNNs are good at learning the relations between inputs and outputs after seeing images several times, there are some complexities of this classification task when using camera imagery. First, extracting visibility features from an image is too difficult. To generalize deep features and understand input image scenes, convolutional layers take advantage of local filters by convolving the entire input space. This is very convenient in a task of classification and object detection. In the case of visibility estimation, it can be used for estimating appropriate visibility distances on the input image, selecting prominent objects as the point of reference. Nevertheless, it is not always possible to find a suitable object in the desired direction.

To the best of our knowledge, no model that estimates visibility from camera imagery by adapting different visibility ranges has been proposed so far. This is because visibility features appear strongly different even when they belong to the same class. This occurs mainly in clear weather condition images, whereas low-visibility images within adjacent classes are strongly similar; hence, the gap between the visibility values is required to be greater to make an accurate prediction, which is another difficulty for the classifier. Therefore, when it comes to evaluating unseen images, the network predicts the wrong answer. These cases occur too frequently and extracting the link between input images and visibility values becomes too challenging.

**Figure 1:**
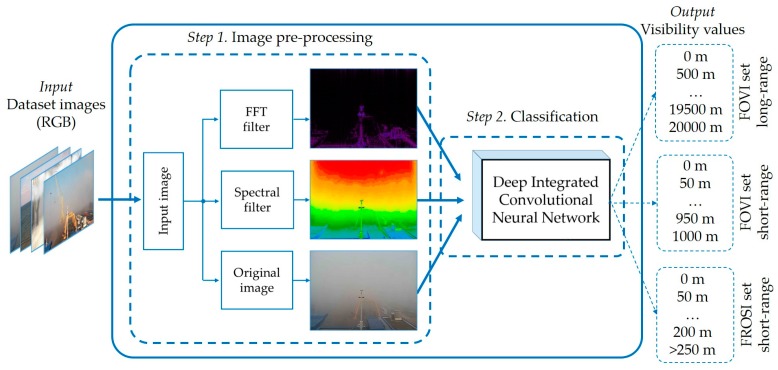
Structure of the VisNet that can adapt different visibility ranges. The model has two stages: pre-processing and classification.

To tackle these complexities, we propose the idea of learning dissimilar features of the same input image simultaneously. Our initial practices showed that in the case of visibility estimation, a small change in the input image strongly affects the future outcomes. This behavior forced us to perform extensive experiments and led us to the most optimal preprocessed input types as well as a novel neural network architecture. The VisNet comprises two steps: First, the model receives images from the dataset and processes each image to reveal the hidden features that help the network to understand the visibility level. Then, a deep integrated CNN simultaneously learns three appearances of the given input and produces the classification output. The working principle of the VisNet is shown in Figure 1.

Initially, an input image is filtered in the frequency domain to remove low frequencies (non-sharp features, such as fog, cloud, and quite sea) from the image. This process is performed using the high-pass filter relying on the fast Fourier transform (FFT) algorithm [97].

The output of this process is mainly edges, curves, and other sharpness features of the given image. The key reason for employing FFT-based filtering is that visible features near the camera will be more strongly visible than those located too far away. Compared to the edge detection algorithms, this approach is very reliable. As edge detection algorithms do not account for the resulting pixel intensities, they extract undesired edges and false lines or even worse, and simply ignore some features because essential information might be lost during processing. Subsequently, a spectral filter is applied to the input image to obtain the regions of fog and low contrast. This stage enables the classifier to learn features that are more global. In other words, the second image type mostly extracts the fog itself, while the first type extracts objects on the image. The last input type is the original input image that is trained in parallel with the second image type.

As it is depicted in Figure 1, the model was trained with three different datasets (more details in Section 3.2. Datasets). By using multiple datasets, we evaluated the performance of the VisNet in various types of images as well as different visibility ranges. Initially, we focused on very long distances (more than 20 km). Afterwards, short-range visibility images were experimented with (up to 1 km). Lastly, very short visibility images (up to 250 m and more) were trained and experimented. It achieved a high performance and confidently classified clear weather as well as dense fog captured images thanks to the image pre-processing step. The classifier based on a deep integrated CNN makes the estimator more robust and accurate. The input data, Steps 1 and 2, and outputs in Figure 1 are described in the following sections.

### 3.2. Datasets

Owing to the difficulty of collecting sufficient foggy images with annotations, there are only a few datasets available concerning the visibility problem. Nevertheless, their data formats, image sizes, and variations are not satisfactory. Therefore, gathering a proper dataset was the first priority task for us. To evaluate the effectiveness of the proposed method and verify it with real-world images, we collected a new set of images using common CCTV cameras. Using the collected dataset, we also evaluated the previously proposed models. Furthermore, to deeply analyze the performance of the model, as well as to describe a fair benchmark, we used an existing small-size FROSI dataset, which is broadly used for visibility and fog phenomena. This, in turn, should reveal the superiorities of the proposed approach compared to the previous state-of-the-art method. All datasets are explained below in detail.

**Figure 2:**
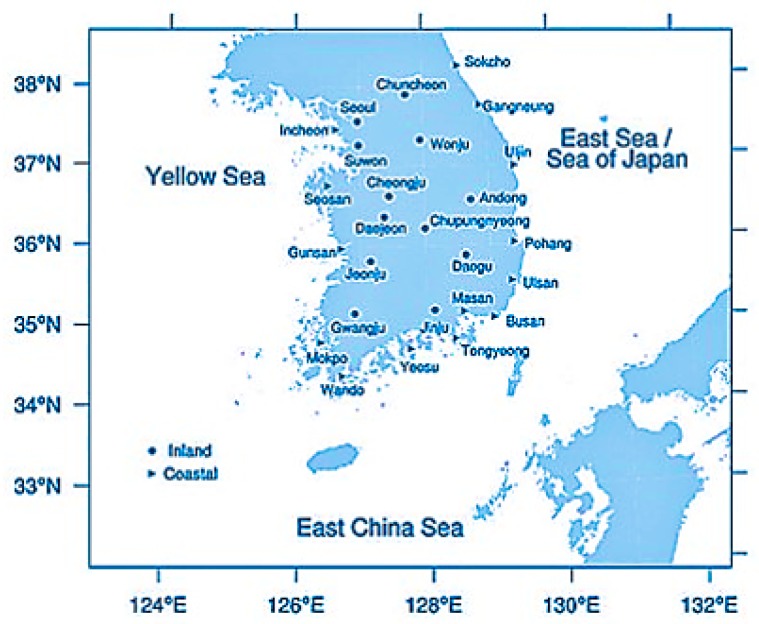
The map of stations for the visibility observation (from Ref. [98]).

In Ref. [98], different characteristics of fog occurrence over the Korean Peninsula and their analysis were presented. According to them, a total of 26 observation stations, shown in Figure 2, of the South Korea Meteorological Administration have been analyzed regarding poor visibility cases based on the data collected over the last 20 years. Based on the statistics, we identified our target locations to collect images. We focused on the regions with a high frequency of fog occurrence, high fog density, different mist and low-cloud occurrences in four seasons, and both coastal and island and urban and clear sea regions.

**Figure 3:**
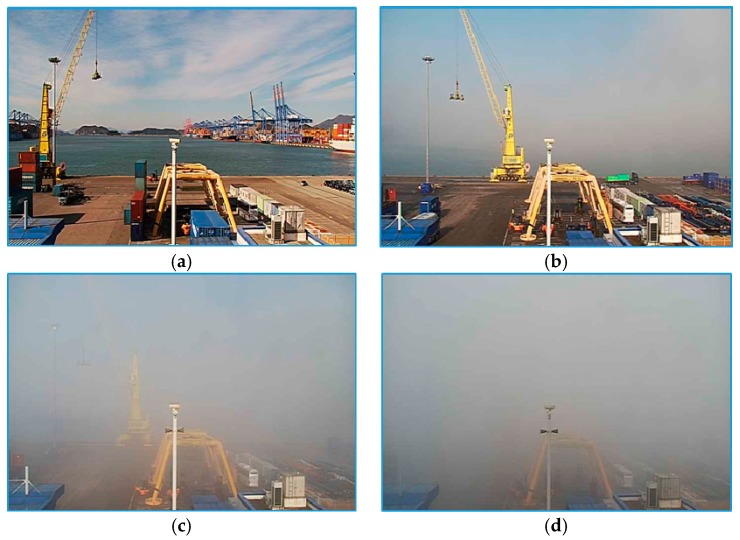
Samples from the FOVI dataset. (**a**) Very good visibility; (**b**) poor visibility (less than 2 km); (**c**) very poor visibility (less than 1 km); (**d**) dense fog (less than 50 m).

**Figure A1:**
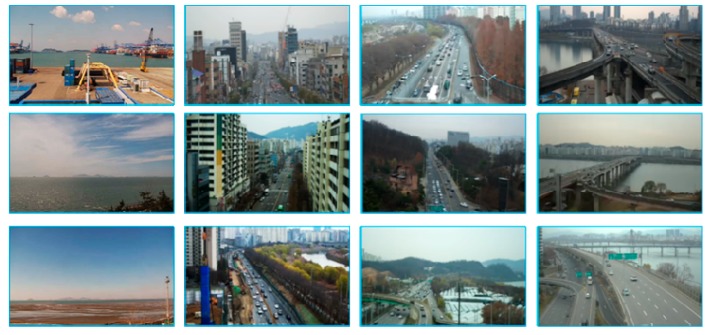
Samples from the collected FOVI dataset. Each image saved in the RGB color space with a resolution of 704 × 576. Visibility values registered as a text file with a range of 0 to 20,000 m.

We decided to use only daytime images (from 8 am to 5 pm) because of the poor image quality during night, and hence, removed images captured during the nighttime, including those with different anomalies, such as images with a dirty lens, strong storm or rain, and heavy snow. As a result, we had around 3 million selected images, each with its exact visibility value. Because the VisNet classifies outdoor imageries, FOVI consisted of outdoor scenes, such as buildings, bridges, sea, mountains, and port views; some examples are given in Figure A1 (see Appendix A). From all collected images, a limited number of images were selected. To evaluate our model’s capability for different visibility ranges, we prepared two datasets for short- and long-range images. Table 2 summarizes the distribution of images between classes, and Figure 3 illustrates the examples of single-camera images obtained from four different classes. 

**Figure 4:**
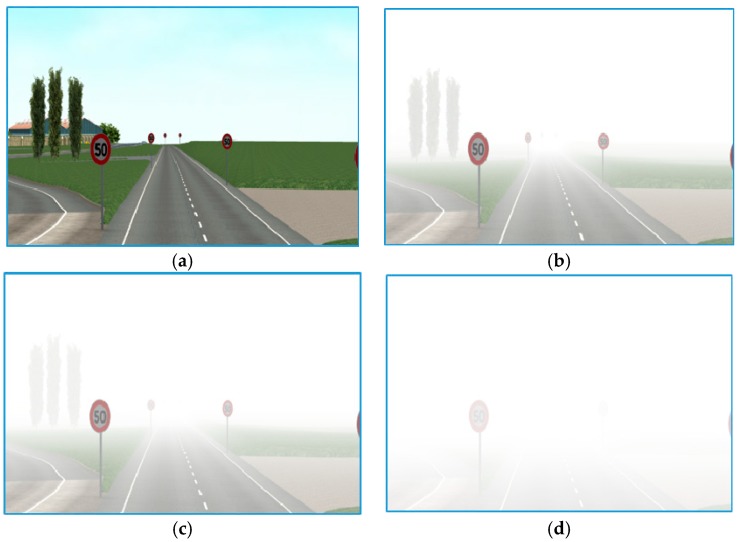
Samples from the FROSI dataset. (**a**) Excellent visibility; (**b**) less than 250 m, (**c**) less than 150 m; (**d**) less than 50 m.

The second dataset, FROSI, is a standard set of synthetic images used for evaluating a systematic performance of road sign detectors in dissimilar foggy situations [52]. Another use of the dataset is for the evaluation of visibility estimators as provided by the previous state-of-the-art approach [63]. For each image, a set of seven types of uniform fog produced with the visibility distances ranging from 50 m to above 250 m [52], resulting in 3528 images, was used. Figure 4 presents different appearances of the same image from four different classes of the FROSI dataset.

### 3.3. Image Pre-Processing

#### 3.3.1. FFT-Based Filter

**Figure 5:**
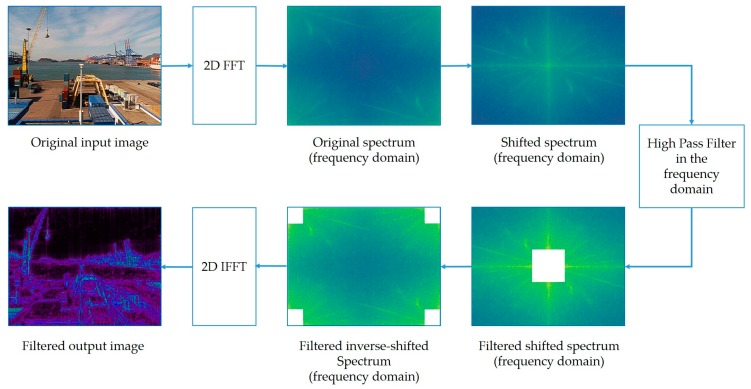
2D fast Fourier transform based filtering algorithm. The block-diagrams 2D FFT and 2D IFFT are fast Fourier transform and inverse fast Fourier transform accordingly. 2D FFT converts the image to frequency domain, 2D IFFT converts the filtered image into spatial domain from the Fourier domain. The high pass filter filters the spectrum in the frequency domain and removes low frequencies.

Figure 5 shows a two-dimensional (2D) FFT-based filtering algorithm, which is a practical important tool in the field of image processing [97,99]. It is used in different areas of applications, such as image analysis, filtering, compression, and reconstruction [97]. However, this is the first time such filtered input images have been used for the deep CNN model to estimate visibility. The key reason for using the 2D FFT algorithm is that it efficiently extracts features based on the frequency of each pixel in the image. In other words, it filters the input image in the frequency domain, rather than the spatial domain. Compared to the other methods, it is very sensitive and can detect even very small changes in frequencies. Because of being computationally intensive, FFT indispensable algorithms have been used in digital signal processing for most real-time applications and image comparison problems, such as advanced fingerprint scanners [100]. Another important reason for using this algorithm is that in clear weather conditions, a captured scene or features of the input image will always dynamically change (sometimes an image of the crowded road and sometimes a view of the clear sea or sky). It is unlikely that only features located near a camera are visible in the heterogeneous foggy weather, and the rest are occupied with fog, and thus, are mostly invisible. Eventually, the model will face challenges while extracting useful features. If we want to predict visibility using the same model, at least for these two cases, it must distinguish useful features from all input images. The image is in the spatial domain, but after transformation, the output will represent the image in the frequency or Fourier domain. In the Fourier domain, a linear combination of harmonic sine and cosine functions is decomposed to the image function of *f* (*m*, *n*). Each point of the image in the frequency domain represents particular frequencies contained in the spatial domain image. The number of frequencies corresponds to that of the pixels in the spatial domain image [101]. The 2D FFT contains only a set of frequencies forming an image that is large enough for a full description of the spatial domain image. Usually, a 2D transformation takes a complex array. Similarly, image processing is mostly applied where each value in the array represents pixel intensities (real numbers), so the real value is the pixel value and the imaginary value is zero [102]. To perform FFT, an image should transform such that the width and height are an integer power of two. It is usually obtained by 0-pad to the nearest integer power of two [102,103,104]. The 2D FFT of an *M* × *N* image can be defined as:

(1) $$F(u,v)=\frac{1}{MN}\sum_{m=0}^{M-1}\sum_{n=0}^{N-1}f(m,n)e^{-2\pi i(\frac{mu}{M}+\frac{nv}{N})}$$

where *f* (*m*, *n*) is the image function; *u*, *v* are the spatial frequencies; and the exponential term forms the basis function corresponding to each point, *F* (*u*, *v*), in the Fourier space. *M* and *N* indicate the width and height of the image accordingly. The basis functions are usually sine and cosine waves with increasing frequencies, *F* (0, 0) represents the DC (Direct Current) component or the mean of the input image that corresponds to the average brightness, and *F* (*M* − 1, *N* − 1) represents the highest frequency. In the case of efficient computation, we can use the separability property of Equation (1) by rewriting it as Equation (2):

(2) $$F(u,v)=\frac{1}{MN}\sum_{m=0}^{M-1}\sum_{n=0}^{N-1}f(m,n)e^{-2\pi i(\frac{mu}{M}+\frac{nv}{N})}=\frac{1}{M}\sum_{m=0}^{M-1}e^{-2\pi \frac{mu}{M}}(\frac{1}{N}\sum_{n=0}^{N-1}f(m,n)e^{-2\pi i\frac{nu}{N}}).$$

Now, the process of computing is divided into two parts, where only one-dimensional (1D) FFT is involved. Thus, for implementing 2D FFT, we will use 1D routines:

1. For each row of *f* (*m*, *n*), performing a 1D FFT for each value of m;
2. For each column, on the resulting values, performing a 1D FFT in the opposite direction.

The transformed image can be re-transformed to the spatial domain using Equation (3). Where *f* (*m*, *n*) is a linear combination of harmonic functions of the basis term and *F* (*u*, *v*) indicates the weights of the harmonic components in the linear combination (complex spectrum):

(3) $$f(m,n)=\sum \sum f(u,v)e^{-2\pi i(\frac{mu}{M}+\frac{nv}{N})}$$

**Figure 6:**
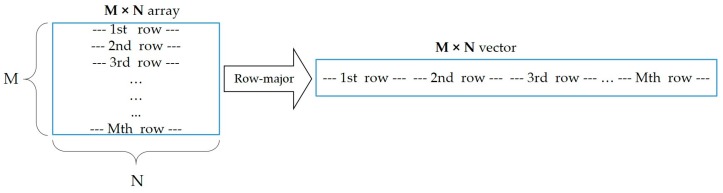
Logical implementation of the row-major format.

From the practical standpoint, let us assume to have an *M* × *N* grayscale image composed of an *M × N* pixel matrix. To perform the above process, we will simply put all rows of the matrix one after another (*M* rows *N* times), which will result in an *M × N* vector. This is known as a row-major format [97], as shown in Figure 6. Since we have our data in the row-major format, the vector has *M × N* entries. We will apply 1D transforms first to all rows and then to all columns. This will incur:

1. *M* 1D transforms over rows (each row having *N* instances);
2. *N* 1D transforms over columns (each column having *M* instances).

**Figure 7:**
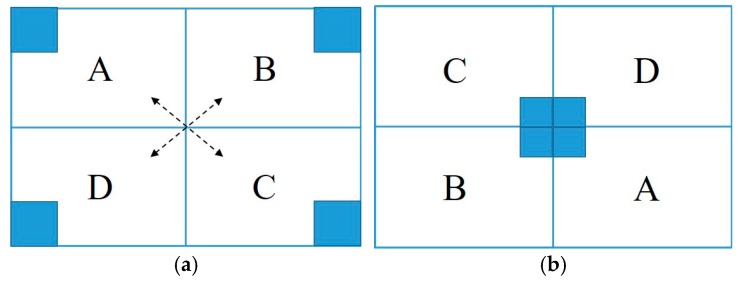
Shifting the DC value (Direct Current component) in the center of the image. (**a**) Original spectrum; (**b**) shifted spectrum.

Usually, during the process, shifting the Fourier image is necessary because a point further away from the center of its corresponding frequency will be higher, so we place the zero-frequency component at the center of the spectrum. We display the *F* (0, 0) or DC-value in the center of the image depicted in Figure 7.

**Figure 8:**
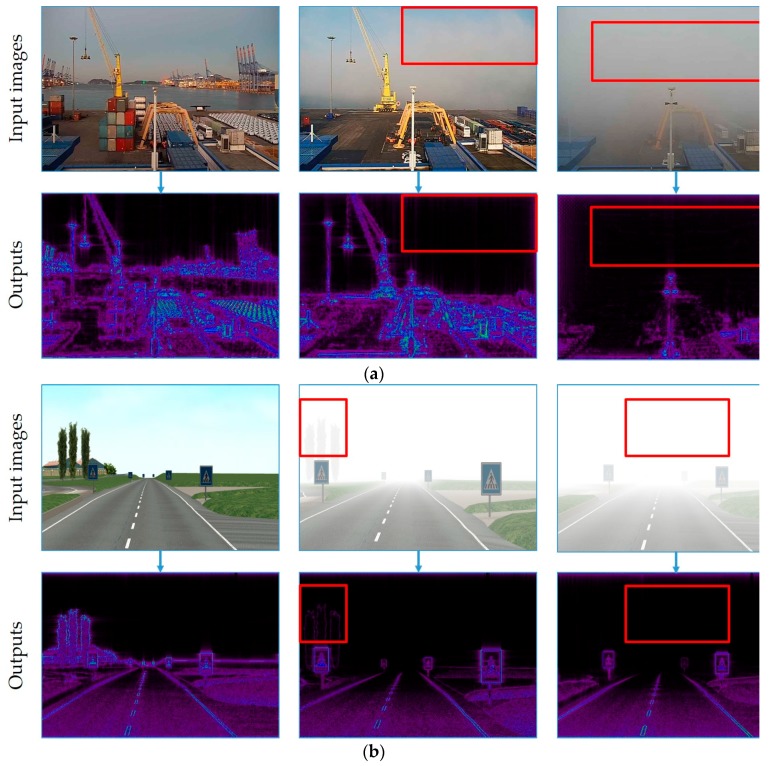
Outputs of the FFT-based filtering algorithm. Cloudy regions of the input image removed successfully (red rectangle). (**a**) Results from FVOI dataset; (**b**) results from FROSI dataset.

Figure 7 clearly shows that the original spectrum can be divided into quarters, but interesting information is always available in the corners (small gray-filled squares represent positions of low frequencies). Due to their symmetric positions, we can swap them diagonally such that the low frequencies appear in the middle of the image [105]. Inverse shifting is the reverse process of shifting. It is also essential to obtain the image back; we shifted the image such that the zero-frequency component is in the center of the spectrum to obtain the previous output. To shift it back, the same process of shifting is performed, but in reverse order. During this process, basically, three different types of filters (low-pass, high-pass, and band-pass filters) are used with the Fourier image [106]. The low-pass filter removes high frequencies, while the low frequencies remain unchanged. This filtering is equivalent to the application of smoothing filters in the spatial domain. On the other hand, the high-pass filter attenuates low frequencies resulting in the spatial domain and yields edge enhancement or edge detection, because edges usually contain many high frequencies. The band-pass filter is useful for enhancing edges for the removal of very low and very high frequencies, but retains a middle range band of frequencies [101]. After FFT is applied to the input image and shifted, we can perform a filtering operation in the frequency domain. We aim to obtain frequencies higher than the given threshold, and therefore, we use a high-pass filter to remove low frequencies. In practice, since we obtain the output image after shifting, which is a complex array attenuation, it is performed using masking with a rectangular window (kernel) of a size of 50 × 50 pixels. The size of the kernel is the threshold, which is capable of blocking high frequencies (mostly edges) and removing the remaining low frequencies by convolving. We can see that most of the image data are present in the low-frequency regions of the spectrum in Figure 5. Finally, the high-pass filter maintained only pixels that correspond to the high frequency, and the results are presented in Figure 8. 

The features located close to the camera appear to be stronger than those located far, which will be very convenient to later evaluate the visibility using the classifier. Most importantly, using the proposed filtering algorithm can remove most of low-frequency features, including cloud, fog, and quiet sea, from the image, so only highly visible features and objects will be evaluated (Figure 8).

#### 3.3.2. Spectral Filter

**Figure 9:**
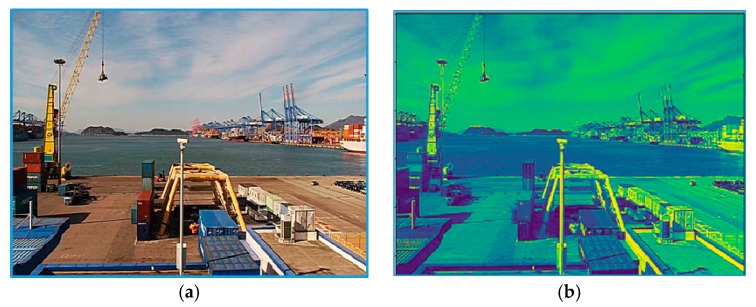

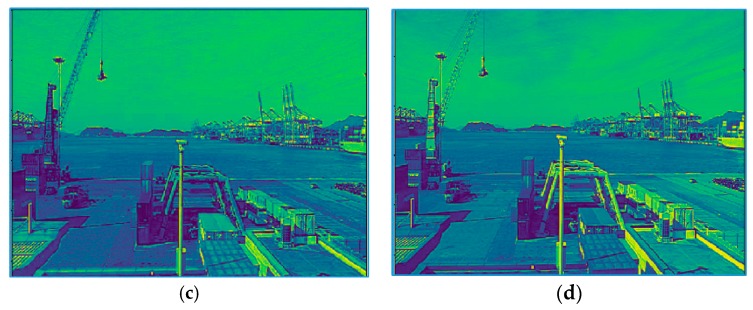
Layers of the RGB color channel of an input image. (**a**) An original input image; (**b**) B-channel (blue); (**c**) R-channel (red); (**d**) G-channel (green).

The contrast of an image taken in dense fog is usually very low, and hence, presents poorly visible scenes [32]. Fog is visible and dominant, but object features are hidden and abstract. In this situation, most CNN-based classifiers struggle most of the time, as they always look for low-level features (such as edges and different shapes) and cannot evaluate visibility. This is because, instead of learning the whole (global) scene, CNNs tend to mostly learn local features. Usually, fully connected layers are added on top of convolutional layers to learn global features after convolutional layers. Nevertheless, new foggy images easily fool the network. Therefore, we try to mimic the visual perception and evaluation process of humans, because our visual system is very sensitive to colors rather than grayscale [107]. To analyze the model performance, we implemented different baseline CNN models to train original images as well as filtered images. We used the same training environment and parameters. Several experiments revealed that the spectral filtered images used in all models achieved better performance than original foggy images. This is because filtered images have more features than original ones. Thus, we can say that CNNs are more sensitive to colorful images than low-contrast images. Images of dense fog are very close to grayscale images due to the low contrast. Therefore, to extract fog from an image, we need to apply a spectral filter to extract the fog effect. The proposed filtering algorithm uses pseudocoloring, as we want to enhance the contrast and other hidden information from the image. A color image can easily be converted to grayscale. However, we need to use a virtual color or color fusion to convert a black-and-white image to a color image [108]. One efficient method to achieve this is pseudocoloring. It is useful when the contrast is quite poor, especially in an image of low visibility. The technique easily enhances the contrast because the pseudocolor is only relevant to single-channel (luminosity) images, and contains abundant information [109]. If there is a small difference between the intensity of the adjacent pixels, a human cannot extract all information. This anomaly also affects CNNs because images that illustrate dense fog do not contain much information. Therefore, the details of images will be more explicit and targets will be more easily recognized when they are transformed to pseudocolor images. Currently, we have an image in the RGB color space; each pixel in the image has three values for each RGB band (RGB stands for red, green, and blue, respectively). The RGB form of the image composed of a 3D color grid corresponds to each band. In other words, there are three grayscale layers of the image, which merge to form the color image. One can choose an RGB channel from the image as all color bands of the RGB are very similar (Figure 9).

**Figure 10:**
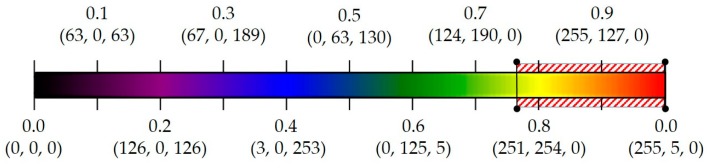
Spectrum of our color map. Fog and low contrast mostly appear in the color within the red-dashed range.

Because the blue channel includes more data relevant to contrast, we will select the blue channel as the desired image, instead of any random one. A common use case that involves a similar pseudocoloring technique is during scientific visualization of flat 2D surfaces, such as geographic fields, dense arrays, or matrices [108]. Sometimes, these obtained images might initially be unrecognizable, so some preprocessing steps are required that affect the images analysis, e.g., noise removal and contrast enhancement. The solution in this case is a color map, which is a continuum of colors that map linearly to a range of numeric values. Usually, the “rainbow” is a default tool to represent data in meteorology [110,111]. The common rainbow is where values between −1 and 0 map to blue colors and those between 0 and 1 map to red colors of varying brightness [110,112,113]. Generally, blue is mapped to the lowest value and red to the highest. Other data values are interpolated along the full spectrum. Although the existing rainbow colors are widely used, they have some problems, such as irregular perception [110], non-uniformity [110,112], and sensitivities to color deficiencies and unnatural ordering [110,112,114]. Thus, we have re-construction [115], which is more suitable for this task. We reduced the number of colors in the spectrum and made the colors uniform. Six colors are included in our revised version: Black, purple, blue, green, yellow, and red, where black represents the lowest value, while red indicates the highest. The colors are scaled in the range of 0 to 1 and are linearly segmented. The proposed spectral color map and values of the RGB components are presented in Figure 10.

**Figure 11:**
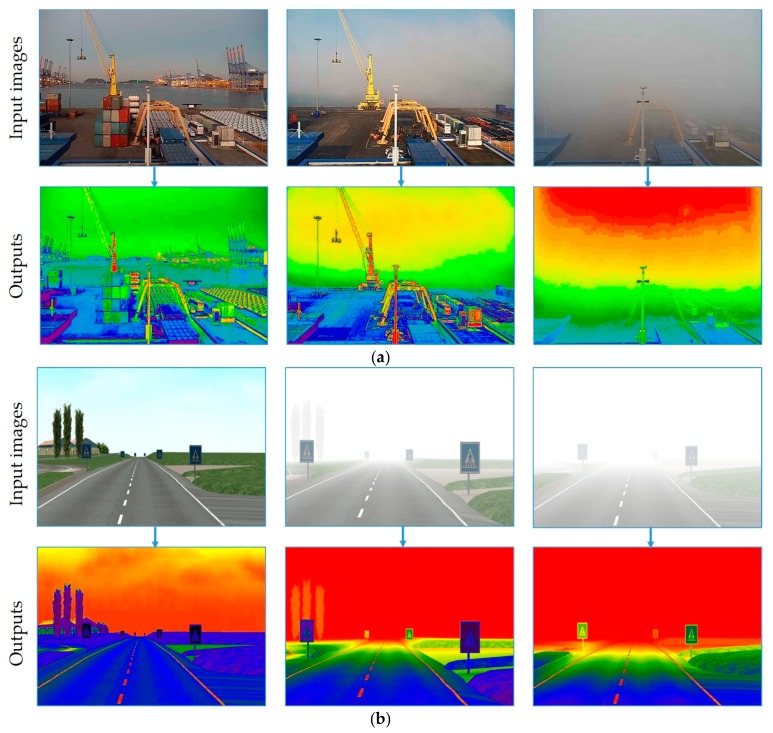
Outputs of the spectral filter. (**a**) Results from the FOVI dataset; (**b**) results from the FROSI dataset.

Re-construction of [115] was accomplished by creating a dictionary that specifies all changes in the RGB channels from one point to the other end of the color map. Let us give the notation, (*x*, *y*_0_, *y*_1_), to each R, G, and B component; in other words, the tuple, (*x*, *y*_0_, *y*_1_), is the entry for each given color. As shown in Figure 9, there are discontinuities in the spectrum and we want to change each color from *y*_0_ to *y*_1_. The last two elements of the tuple, (*y*_0_, *y*_1_), are the starting and ending points and the first element, *x*, is the interpolation interval throughout the full range (from *y*_0_ to *y*_1_). At each step *i* between *x*[*i*] and *x*[*i* + 1], the value of the color is interpolated between *y*_1_[*i*] and *y*_0_[*i* + 1]. Each color value increases from *y*_0_ to *y*_1_, and then jumps down. For this case, values of *y*_0_ [0] and *y*_1_[−1] are never used, and for the *x* value, the interpolation is always between *y*_1_[*i*] and *y*_0_[*i* + 1]. The final obtained results of the spectral filtering algorithm are given in Figure 11. We apply the color map after the pseudocoloring and each pixel gains new values, which present a clearer image that is easy to detect fog and has low contrast. As shown in Figure 11 (bottom), distinguishing different foggy images from the original one is much easier for human visual perception. Similarly, the performance of the classifier is higher than that of training original images. The experiment results are given in Section 4.

### 3.4. Classification

**Figure 12:**
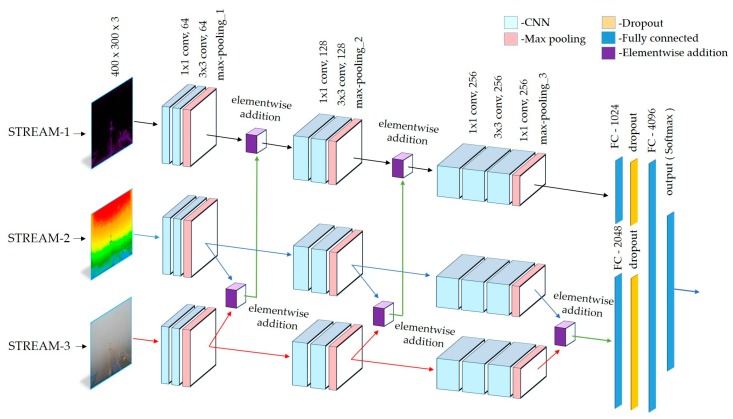
The architecture of the deep integrated CNNs.

As shown in Table 1, the task of classification in this study involves distinguishing 41, 21, and 7 classes of images using a single model described in Figure 1. To accomplish the classification, we implemented a deep integrated CNN, which was itself designed to adapt to different visibility conditions and ranges by employing three streams of CNNs, as depicted in Figure 12.

These streams are connected in a way that provides robustness to the whole network during the classification. It receives three preprocessed input images, classifies them, and produces a single visibility value, which corresponds to one of the three given visibility ranges. The best performing CNN stream is composed of five convolutional layers followed by two fully connected layers. To extract deep features, the utilization of small-size convolutional kernels is an essential factor. The structure of the network is given in Table 3. Inputs to the first layer are fixed to 400 × 300 × 3 pixels (width × height × channel), and all samples are wrapped to the corresponding size. The first and second convolutional layers of each of the three streams (STREAM-1, STREAM-2, and STREAM-3) utilize 64 filters of a size of 1 × 1 and 3 × 3, which give 64 feature maps by using a stride of 1 × 1 pixels in the horizontal and vertical directions, respectively. 1 × 1 convolution filters are used because they can be a linear transformation of the inputs.

The size of the feature map is 400 × 300 × 64 in the first and second convolutional layers of all three streams. The size of the feature map, in other words, an output of the convolution for the input size of (width × height) and a filter size of (*F_width_* × *F_height_*) is calculated as shown in Equation (4) [116]. Where *S_width_* and *S_height_* are the stride and P is the padding (it is equal to 0 in our network):

(4) $$\begin{array}{c} Output_{Width}=\frac{width-F_{width}+2P}{S_{width}}+1\\ Output_{Height}=\frac{height-F_{height}+2P}{S_{height}}+1 \end{array}$$

In this study, we use the rectified linear unit (ReLU) as an activation function in the form shown in Equation (5) [117]. ReLU has a faster processing speed compared to other non-linear activation functions and can reduce the vanishing gradient problem that might occur in back-propagation during training. Where *x* and *y* are the input and output values, respectively:

(5) y=max(0, x)

Max-pooling is applied to the outputs of the second convolutional layer, and the maximum value is computed with stride 2 in 2 × 2 windows. In the max-pooling layers, feature maps are divided into a set of non-overlapping square sections, and for each such section, the maximum value is calculated. After applying max-pooling, the outputs from STREAM-2 and STREAM-3 propagate to the third convolution layer of the corresponding streams. Hence, the same outputs are elementwise added together. Subsequently, the addition result is elementwise added to the output of the max-pooling layer in STREAM-1 and the resulting inputs are propagated forward. To elementwise add feature maps, each matrix must have an equal number of rows and columns. Addition is performed by simply adding the corresponding elements of each matrix, as shown in Equation (6).

(6) $$\begin{array}{c} A_{m\times n}=\left|\begin{array}{ccc} a_{1,1} & \cdots & a_{1\times n}\\ \vdots & \ddots & \vdots \\ a_{m\times 1} & \cdots & a_{m\times n} \end{array}\right|,\;B_{m\times n}=\left|\begin{array}{ccc} b_{1,1} & \cdots & b_{1\times n}\\ \vdots & \ddots & \vdots \\ b_{m\times 1} & \cdots & b_{m\times n} \end{array}\right|\\ A_{m\times n}+B_{m\times n}=\left|\begin{array}{ccc} a_{1,1}+b_{1,1} & \cdots & a_{1\times n}+b_{1\times n}\\ \vdots & \ddots & \vdots \\ a_{m\times 1}+b_{m\times 1} & \cdots & a_{m\times n}+b_{m\times n} \end{array}\right| \end{array}$$

where *A_m_*_×_*_n_* and *B_m_*_×_*_n_* are *m × n* matrices, and *a* and *b* are the elements of the *A_m×n_* and *B_m__×__n_* matrices. The elementwise addition is described by the sum, *A_m×n_ + B_m×n_*.

The third and fourth convolutional layers of the three streams have the same filter size and stride as the previous layers, i.e., (1 × 1 and 3 × 3) and (1 × 1), respectively. The size of the feature maps, 199 × 149 × 128, is obtained using filters with a size of 128. Similarly, max-pooling is applied to the outputs of the fourth convolutional layer with the same parameters as previously, 2 × 2 kernel size, and stride 2, and the size of the feature maps is decreased to 98 × 73. Elementwise addition is applied to the outputs of the max-pooling layers of STREAM-2 and STREAM-3 prior to adding the outputs of the max-pooling layer in STREAM-1. Finally, the fifth, sixth, and seventh convolutional layers of each of the three streams use 256 filters of sizes 1 × 1, 1 × 1, and 3 × 3 with the strides of 1 × 1, 2 × 2, and 1 × 1, respectively. There are 256 feature maps and the size of each map is equal to 48 × 36 × 256 in the three layers of all three streams. After the application of max-pooling, 256 feature maps with a size of 24 × 18 pixels are obtained through these seven convolutional layers of all streams. 

From Figure 12 and Table 3, the last layers of the proposed network are four fully connected layers, which are also known as traditional MLPs (Multilayer Perceptron) comprising a hidden layer and logistic regression. The first fully connected layer has 1024 neurons connected to the outputs of the last max-pooling layer of STREAM-1. The resulting feature maps of STREAM-2 and STREAM-3 are elementwise added and fed to a separate fully connected layer that has 2048 neurons. As described by [64,118], CNN-based models have an overfitting problem that causes a low classification accuracy with testing data, notwithstanding that the training accuracy is high. To prevent the overfitting problem, we used the dropout methods [64,118]. For the dropout, we set a dropout rate of 40% to randomly disconnect the neurons between the previous layer and the next layers in the fully connected layers. Dropout layers were sandwiched between the first and third fully connected layers, as described in Figure 12. Outputs of the dropout layers are forwarded to the next fully connected layer, which comprises 4096 neurons.

Finally, outputs from 4096 neurons are fed into the final classification layer, located at the top of the network. In the last fully connected layer, a softmax function is used, as shown in Equation (7), to determine the classification results. In addition, the softmax normalizes the sum of the activation values to be equal to 1. This enables the interpretation of outputs as probability estimates of the class membership. Based on Equation (7), the probability of neurons belonging to the *j*th class is calculated by dividing the value of the *j*th element by the sum of the values of all elements [119]. Where *σ*(*s*) is the array of output neurons and K is the total number of classes.

(7) $$\sigma (s)=\frac{e^{sj}}{\sum_{n=1}^{K}e^{sn}}$$

To evaluate proposed model’s performance and convergence more comprehensively, the experimental results are judged by calculating the mean square error (MSE) [81]. The MSE is defined as Equation (8), where ${\hat{V}}_{i}$ is the predicted visibility value, $V_{i}$ is the actual visibility value, and *n* is the total number of used samples:

(8) $$MSE=\frac{1}{n}\sum_{i=1}^{n}{\left|{\hat{V}}_{i}-V_{i}\right|}^{2}$$

## 4. Experimental Results and Discussion

To analyze the performance of the purposed VisNet, we conducted extensive experiments on three datasets, each with a different number of classes. As an implementation tool, a currently popular Tensorflow machine learning library was adopted. Table 4 lists the characteristics of the hardware and the software of the machine. During training, all layers of the entire network were adjusted using the Adam optimizer [120] with a learning rate of 0.00001. Cross-entropy was introduced as a loss function and optimized such that the loss function fitted the one-hot distribution as well as the uniform distribution.

**Figure 13:**
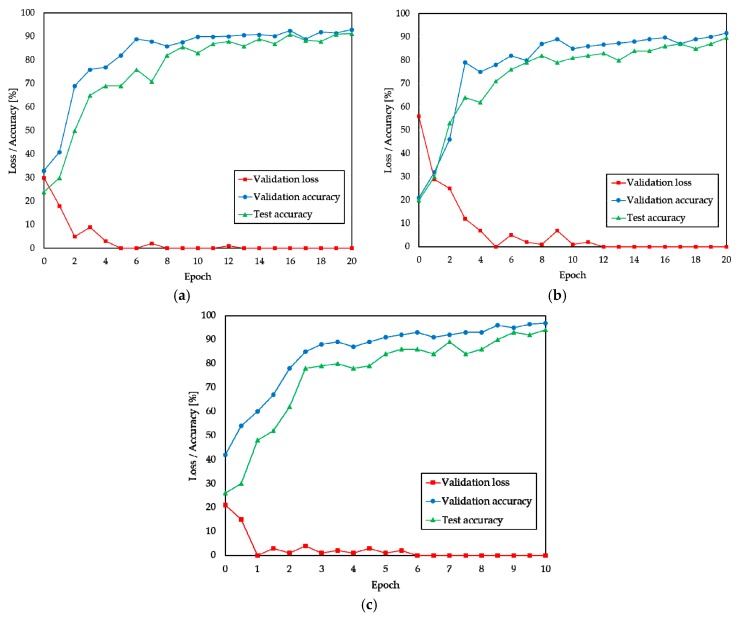
Functions of validation and test accuracy as well as validation loss on three datasets. (**a**) Long-range visibility on FOVI, (**b**) short-range visibility on FOVI, and (**c**) short-range visibility on FROSI datasets.

All three sets of images were split into 70%, 10%, and 20% sets for training, validation, and test, respectively. Since the FROSI dataset comprises few images, we set the number of epochs to 10 to avoid overlooking the training set. The remaining two datasets trained over 20 epochs each. Figure 13a,b show the loss function of validation, accuracy functions of validation, and testing of the long- and short-range images in the FOVI dataset, respectively. Furthermore, validation loss, validation accuracy, and test accuracy functions on the FROSI dataset are depicted in Figure 13c. Table 5 shows the performance of the model after validation and testing for long- and short-range visibility estimation on the FOVI dataset as well as short-range visibility estimation on the FROSI dataset. In Figure 13a, the long-range classification results on FOVI are given, where the validation loss decreases rapidly at the beginning epochs, and after 13 epochs, it reaches its minimum value of 0.37 and maintains a steady index over the course, with a corresponding validation accuracy of 90%. The final validation and testing accuracy are 93.04% and 91.30%, respectively (Table 5). The short-range visibility estimation on FOVI obtains a 91.77% validation and 89.51% testing accuracy. Similarly, the validation loss gains the minimum value of 1.17 after the 13th epoch and maintains a stable rate during validation. Table 5 shows that the VisNet reveals considerable improvements on the FROSI dataset and achieves a 94.03% successful classification rate compared to a 90.2% accuracy, which was obtained by a previously proposed state-of-the-art model [63]. In addition, Figure 13 and Table 5 indicate that the VisNet can be used as a universal visibility estimator as it can distinguish the visibility features of dissimilar input images with different visibility ranges. In other words, increasing the number of streams and connecting them efficiently allows our model to adapt to different visibility ranges and to obtain significant success in visibility estimation from real-world images.

To the best of our knowledge, no model has been proposed so far that can predict the visibility distance of unseen input scenes, especially with different visibility ranges. Moreover, the VisNet obtained a high classification rate, outperforming the other approaches mentioned in Section 2, in visibility estimation from camera imagery. To prove our VisNet’s performance and classification effect, we conducted experiments on three datasets prepared for this study using previously proposed and other well-known models. Table 6 shows a list of models and classification results (using accuracy as the performance indicator). After training, validating, and testing all these models, we compared the experimental results and found that the VisNet is by far most superior to traditional machine learning algorithms and other popular CNN models in visibility estimation from images.

It is important to mention here that the models listed in Table 6 received only original images as inputs to the network. VisNet also receives original images as inputs and processes (applies spectral and FFT filters) prior to training them. Once the model trained, validated, and finished learning the generalized visibility function, the obtained results were applied to the test set images and evaluated error. We used the mean square error (MSE) to evaluate the performance of networks more comprehensively. The final experimental results, the MSE of the validation and test sets of three datasets, are shown in Table 7. The results in Table 6 reveal that the proposed model, VisNet, has a better performance than those of the previously proposed model in the case of visibility estimation. Moreover, the lowest MSE rate was also obtained by our proposed model, see Table 7. Respectively, VGG-16 [66] and Alex-Net [12] achieved the second and third highest performance in classification. Similarly, the second and third lowest MSE was also achieved by VGG-16 [66] and Alex-Net [12].

We need to emphasize here that although the performance of ResNet-50 [65] is high in classification, it showed a low classification accuracy in visibility estimation. We discovered that the reason for the low performance is the number of layers in the network. The model has too many layers that generalize a scene very deeply, and, as a result, it learns too much redundant information instead of learning visibility features. Furthermore, the ResNet-50 [65] is good when input images present the whole scene with different objects, however, images of dense fog with no clear objects confuses the model. This sort of learning leads to strong overfitting and misclassification of the network.

As mentioned earlier, the same CNN model with different input features can have various effects on the same data. Therefore, for evaluation purposes, we separately trained multiple models, listed in Table 6; Table 7, each with original, spectral filtered, and FFT filtered images of the three datasets. Since VisNet consists of three identical streams of CNNs, to evaluate the performance, we decided to train a single stream of CNN with three types of inputs and obtained results combined with the weighted sum rule. The final results are given in Table 8, where ORG is the original images, SPC is the spectral filtered images, and FFT is the FFT filtered images, and the sum of the test results are given in the column Sum. Based on the provided statistics, we can say that VGG-16 [66] achieves the highest classification accuracy on three datasets whereas VisNet’s single stream revealed considerable less accuracy. However, still, the integration of VisNet’s single stream, as depicted in Figure 12, shows the best classification accuracy in contrast to VGG-16 [66]. It is vital to note that within the three different inputs, the classification of the original input images achieved a better performance (using accuracy as the performance indicator) than the remainders. Furthermore, for each network in Table 8, the differences between the three outputs (classification results of three input images) are great, therefore, their sum could not be more than the highest result within these three outputs.

Until now, we evaluated the performance of individual models, but in this stage of the experiments, we ensemble the outputs of multiple models. These experiments improved the performance thanks to the complementarity of the multiple models as described in Ref. [64], which was the top ILSVRC (ImageNet Large Scale Visual Recognition Challenge) submission. From Table 6 and Table 8, it is obvious that VGG-16 [66] achieves the best performance after our VisNet. Since both these networks acquire a strong ability to learn visibility features, we consider the experimenting feature fusion to distinguish visibility features more easily and to be more robust to estimate visibility from camera imagery, resulting in better performance. In these experiments, all CNN models train separately and then fuse the scores using the weighted sum rule after the softmax layer. Such a stacking method allows classifiers to learn how to optimally combine the image statistics over a range of scales and ensure a higher classification accuracy. Table 9 illustrates the accuracy of the fusion models. Models were fused by the weighted sum rule after the softmax layer. In Table 9, the performances obtained by the following ensembles of models are compared:

- VisNet: The proposed model based on deep integrated CNN, which receives original (ORG) input images and processes them using spectral and FFT filters prior to training. Instead of utilizing any decision rule, the CNN streams connected fully-connected layers to integrate streams and finally the softmax layer produces outputs.
- VGG-16/ORG + VisNet: The fusion by the weighted sum rule of the VGG-16 [66] and VisNet. Each model received an original images (ORG) as inputs. VGG-16 [66] was trained using original images only, whereas VisNet receives original images and then processes them for training.
- VGG-16/SPC + VisNet: The fusion by the weighted sum rule of the VGG-16 [66] and VisNet. VGG-16 [66] was trained with spectral filtered images, but VisNet received original images.
- VGG-16/FFT + VisNet: The fusion by the weighted sum rule of VGG-16 [66] and VisNet. The purposed model received original images, the VGG-16 was trained with FFT filtered images.
- VGG-16/ORG + VGG-16/SPC + VGG-16/FFT + VisNet: Weighted sum rule utilized to fuse VGG-16 [66] and VisNet. Three VGG-16 [66] were trained with the same parameters, the only difference was the types of input images, VGG-16 was trained with original, spectral filtered, and FFT filtered images (ORG, SPC, and FFT for each VGG-16). VisNet received original images and processed images before training.

As shown in Table 9, the fusion can obtain slightly better results than those of separately trained models. Nevertheless, this comes at the cost of the increased feature dimensionality. For reference, compared to the VisNet, the classification accuracy improved by just 0.5% and 0.4% (FOVI long-range and FROSI short-range, respectively), but the size of the networks increased significantly due to fusion; as a result, networks have become even more computationally costly. Moreover, the effect of fusion is extremely different for different datasets. For instance, the fusion for VGG-16/ORG + VGG-16/SPC + VGG-16/FFT + VisNet can achieve an obvious effect with a 2.3% increase. However, for VGG-16/ORG + VisNet and VGG-16/FFT + VisNet, the effect of the fusion is not obvious. For these two fusion models, the performance is particularly a good model, which can conceal most of the erroneous outputs of the other model, but its erroneous result cannot be avoided by the other one. Thus, fusion cannot improve the classification accuracy of these models.

Another important finding after fusion is that there is no single model that can obtain the highest performance on three different datasets, see Table 9. Since our main focus is developing a single, robust visibility estimator that can adapt to different visibility ranges, VisNet can be a really efficient model for this task. Hence, Table 6 and Table 8 prove the high classification performance and Table 9 reveals the efficiency and robustness of the VisNet.

As we mentioned earlier, we experimented with different CNN architectures to use them as a stream for the network as connected, as shown in Figure 12. Since all streams in Figure 12 have identical parameters, each implemented a single CNN structure used in all three streams. Experiments were conducted on three datasets. The experimented CNN structures contain three to eight CNN layers. The modifications of the CNN layers are provided in four domains. First, is the number of layers, second is the number of feature maps, third is the kernel size, and fourth is the stride. Changes in the filter size will automatically change the way of scanning the input image. The number of output feature maps is chosen between 16, 32, 64, 128, 226, 512, and 1024. The tested kernel sizes are 1 × 1, 3 × 3, 5 × 5, 7 × 7, 9 × 9, and 11 × 11 for each convolutional layer (C1–C8). We chose the stride according to the filter size from 1 to 5. After many modifications of the organization of the CNN layers, Table 10 investigates the classification results of the five best solutions on three datasets. The best classification solution is named CNN-3 in Table 10.

**Figure 14:**
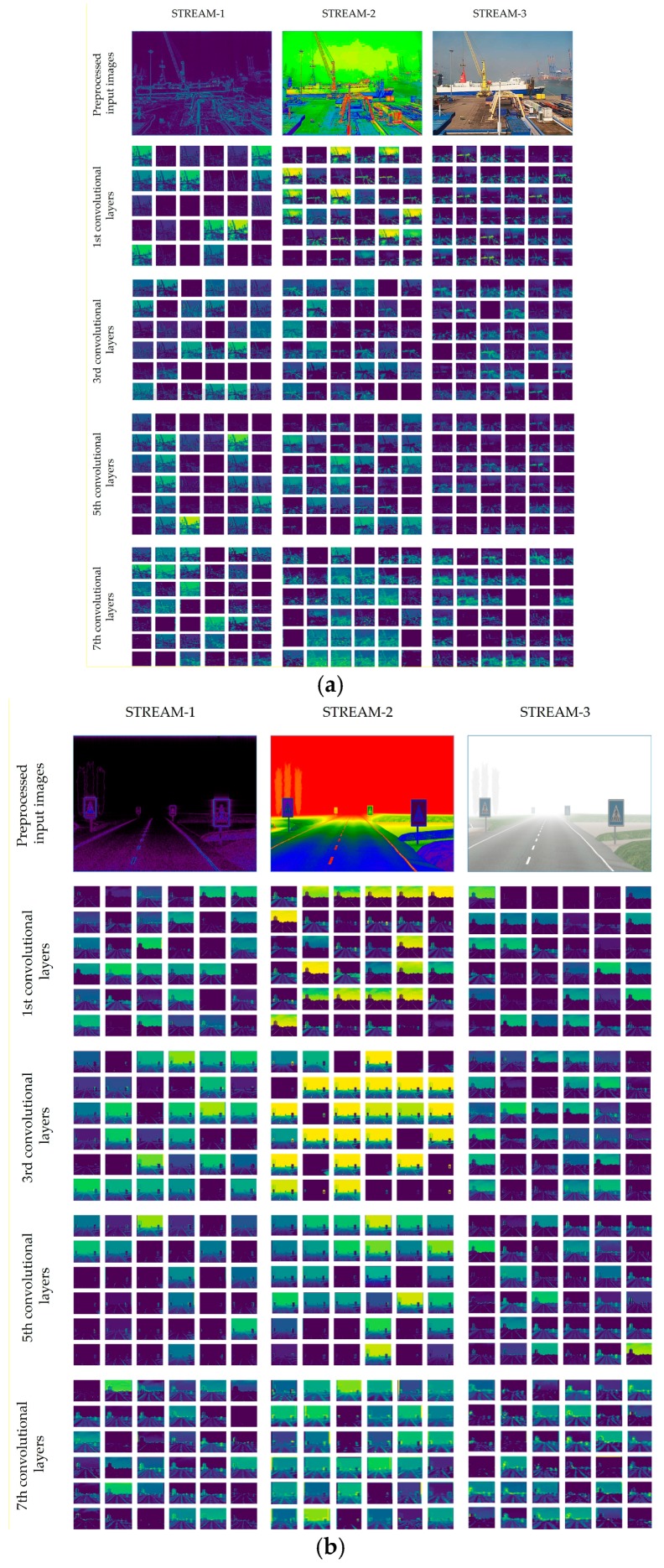
Representations of output filters of three streams. (**a**) Representation of feature maps from the long-range FOVI dataset; (**b**) representation of feature maps from the short-range FROSI dataset.

To deeply analyze the deep integrated network, we visualize some output features of the first, third, fifth, and seventh convolutional layers of all three streams. Figure 14a illustrates the feature maps of the long-range visibility images from the FOVI dataset, while Figure 14b depicts the output features of the FROSI dataset. A single input image was given to the model and pre-processed prior to feeding the CNN streams. To show the behavior of the network, we selected an image that is complex to classify. From the image input to STREAM-3 (original image) in Figure 14a, the area behind the ship is almost invisible due to low cloud, whereas the ship itself is clearly visible. In other words, the ship covers the most important regions of the image and has the essential details (low-visibility features) hidden. Most of the single-stream CNN models fail to predict the visibility distance from this image. The original value of the visibility in this image is equal to 12 km, but all models listed in Table 6 predict a distance longer than 17 km. Our model produced an exact value of visibility. The feature maps of STREAM-1 of both Figure 14a,b are mostly edges and visible objects in the image and convolutional filters mostly learn edges. Scanning STREAM-2 in both figures focuses on the intensity of the pixels that present low contrast (see image input to STREAM-2 in both figures). This is done by applying the spectral filter, which allows low-contrast regions to appear clearer so that convolutional filters detect these regions easily. Scanning the original images allows the network to learn other dissimilar features of the image, including edges and hidden contrast details (STREAM-3).

## 5. Conclusions

Here, we studied a new model, namely VisNet, which was implemented based on a deep integrated CNN to estimate the visibility distance of any outdoor image captured in the daytime. In addition, we collected a huge dataset of images with their corresponding visibility values. To validate and evaluate our model’s performance, we used three different datasets of images, each with different visibility ranges and a different number of classes. Compared to previous approaches, the proposed VisNet was more robust and could easily be used as a universal visibility estimator. During these experiments, we found that to estimate visibility from images, the structure of integrated multiple streams of deep CNNs gives better classification effects than those of the networks with a single stream. Such network architecture adapts different visibility ranges easily and handles both small and large datasets of images by simply controlling the iteration steps. Moreover, employing pre-processing steps, which process each input image before feeding to the deep network, increases the performance of the network considerably.

Moreover, during experiments, we evaluated several well-known models, such as VGG-16, AlexNet, and ResNet-50, and compared them with the proposed VisNet. Our model revealed greater classification performance than those models. In addition, we fused the VisNet and the second best-performed VGG-16; as a result, we obtained a slight improvement. However, the improvement was very low whereas almost double the resources and computation power were used for the fusion than when separately trained. We trained fusion by the weighted sum rule of VGG-16 and VisNet with different input images. As a result, three different models achieved better performance, but we aimed to gain a single best model for several datasets. Therefore, we can conclude that the fusion of VGG-16 and VisNet is very inefficient.

Although our model successfully classifies the visibility images very accurately, there are some limitations. The first drawback is the computation time. Because the network has a pre-processing stage as well as several integrated CNN layers, the training is time-consuming. Another setback is that the model can use only daytime images, as images taken at night have almost no features and classifying them requires a different approach. These limitations will be further investigated in the future. Furthermore, in our future research, we will also focus on reducing the complexity of the network. This, in turn, should decrease the demand for extra resources and training time. Also, the fusion of different CNNs should also be evaluated, as an efficient ensemble of suitable networks would significantly improve the performance.

## Figures and Tables

**Table 1:** Comparisons of previous proposed methods.

Category	Used Methods	Evaluation Range [m]	Advantages	Disadvantages
ANN-based methods	Uses feed-forward neural networks [63] and FROSI dataset [52]	60–250	High evaluation accuracy on the FROSI dataset; Fast evaluation speed;	Classifies only synthetic images and cannot be used for real-world images; Only short visibility range can be estimated;
	Combination of CNN and RNN are used for learning atmospheric visibility [29]. Relative SVM [29] is used on top of CNN-RNN model. Input images collected from the Internet.	300–800	Can effectively adapt to both small and big data scenario;	Computationally costly and consumes long time to train; Uses manual annotations instead of more reliable sensors;
	Pre-trained CNN model (AlexNet [12]) is used to classify webcam weather images [12].	5000–35,000	Can evaluate a dataset of images with smaller resolutions; Faster training because of using cropped patches of input images;	Uses an unbalanced dataset; Test accuracy is very low (61.8%); Reveals too high prediction error (±3 km); Reference objects are required for accurate classification;
	Deep neural networks for visibility forecasting [16]. Uses 24 h a day observation data from 2007 to 2016 (these inputs are temperature, dew point temperature, relative humidity, wind direction, and wind speed.)	0–5000	A working application for aviation meteorological services at the airport;	Achieves a big absolute error (the absolute error of hourly visibility is 706 m. The absolute error is 325 m when visibility ≤1000 m); Several types of input data are required that collected using expensive sensors;
	Visibility distance is evaluated using a system consists of three layers of forward-transfer, back-propagation, and risk neural network [62]. A meteorological dataset, which collected within 5 years, utilized to verify the model.	0–10,000	A higher risk is given to low-visibility and vice-versa; Outperforms standard neural networks and baseline regression models;	Needs a collection of several daily data that require high-cost measurements to predict future hours; Uses only the average values, not real values, of the data; Focuses mostly on low-visibility; Evaluation of high visibility conditions is error-prone; Carefully parameter adjusting is required to avoid biased learning process;
Statistical methods	Estimation of atmospheric visibility distance via ordinary outdoor cameras based on the contrast expectation in the scene [18].	1–5000	Uses publicly available dataset; No need to calibrate the system to evaluate visibility;	Model-driven; More effective for high-visibility; An estimation low-visibility is error-prone; Requires extra meteorological sensors to obtain important values;
	A model based on the Sobel edge detection algorithm and normalized edge extraction method to detect visibility from camera imagery [25].	400–16,000	Uses small amounts of images; Automatic and fast computation;	Cannot predict the distance if visibility is less than 400 m; Uses high-cost camera (COHU 3960 Series environmental camera);
	Uses Gaussian image entropy and piecewise stationary time series analysis algorithms [36]. A region of interest is extracted taking into account the relative ratios of image entropy to improve performance.	0–600	Uses a very big dataset (2,016,000 frames) to verify the model;	Can be used only road scenes; Works effectively only in uniform fog;
	Exploration of visibility estimation from camera imagery [85]. Methods are landmark discrimination using edge detection, contrast reduction between targets, global image features (mean edge, transmission estimation using DCP), decision tree, and regression methods.	250–5000	Uses low-cost cameras; No need camera calibration; Low-resolution images can be evaluated;	Target objects are needed; Different methods require different reference points as targets; Can be used in a fixed camera environment;

**Table 2:** Distributions of images between classes on both datasets.

Dataset	Range	Total Number of Selected Images	Visibility Distance [m]	Number of Classes	Range Between Classes [m]
FOVI	Long-range	140,000	0 to 20,000	41	500
	Short-range	100,000	0 to 1000	21	50
FROSI	Short-range	3528	0 to >250	7	50

**Table 3:** Distributions of images between classes on both datasets.

	STREAM-1		STREAM-2		STREAM-3			
Layer Type	Num. of Filters	Size of Feature Map	Num. of Filters	Size of Feature Map	Num. of Filters	Size of Feature Map	Size of Kernel	Num. of Stride
Image input layer (weight × height × channel)		400 × 300 × 3		400 × 300 × 3		400 × 300 × 3		
1th convolutional layer	64	400 × 300 × 64	64	400 × 300 × 64	64	400 × 300 × 64	1 × 1 × 3	1 × 1
2nd convolutional layer	64	398 × 298 × 64	64	398 × 298 × 64	64	398 × 298 × 64	3 × 3 × 3	1 × 1
Max-pooling layer	1	199 × 149 × 64	1	199 × 149 × 64	1	199 × 149 × 64	2 × 2	2 × 2
			Elementwise addition					
	Elementwise addition							
3rd convolutional layer	128	199 × 149 × 128	128	199 × 149 × 128	128	199 × 149 × 128	1 × 1 × 3	1 × 1
4th convolutional layer	128	197 × 147 × 128	128	197 × 147 × 128	128	197 × 147 × 128	3 × 3 × 3	1 × 1
Max-pooling layer	1	98 × 73 × 128	1	98 × 73 × 128	1	98 × 73 × 128	2 × 2	2 × 2
			Elementwise addition					
	Elementwise addition							
5th convolutional layer	256	98 × 73 × 256	256	98 × 73 × 256	256	98 × 73 × 256	1 × 1 × 3	1 × 1
6nd convolutional layer	256	48 × 36 × 256	256	48 × 36 × 256	256	48 × 36 × 256	3 × 3 × 3	2 × 2
7nd convolutional layer	256	48 × 36 × 256	256	48 × 36 × 256	256	48 × 36 × 256	1 × 1 × 3	1 × 1
Max-pooling layer	1	24 × 18 × 256	1	24 × 18 × 256	1	24 × 18 × 256	2 × 2	2 × 2
			Elementwise addition					
1st and 2nd fully connected layers	1024		2048					
Dropout layers	1024		2048					
3rd fully connected layer	4096							
Classification layer (output layer)	41 or 21 or 7							

**Table 4:** Characteristics of the hardware and software of the machine.

Item	Content
CPU	AMD Ryzen Threadripper 1950X 16-Core Processor
GPU	NVIDIA GeForce GTX 1080 Ti
RAM	64 GB
Operating system	Windows 10
Programming language	Python 3.6
Deep learning library	Tensorflow 1.11
Cuda	cuda 9.2

**Table 5:** Results of the validation and test accuracy on three datasets (unit in %).

Dataset	Range	Validation Accuracy	Test Accuracy
FOVI	Long-range	93.04	91.30
	Short-range	91.77	89.51
FROSI	Short-range	96.80	94.03

**Table 6:** List of models and classification results (unit in %).

Models	FOVI Dataset (Long-Range)		FOVI Dataset (Short-Range)		FROSI Dataset (Short-Range)	
	Val. Acc.	Test Acc.	Val. Acc.	Test Acc.	Val. Acc.	Test Acc.
Simple neural network [63]	48.0	45.2	42.9	38.1	58.8	57.0
Relative SVM [29]	69.7	68.5	59.0	57.0	73.0	72.2
Relative CNN-RNN [29]	82.2	81.3	79.0	78.4	85.5	84.4
ResNet-50 [65]	72.0	70.9	71.8	68.6	82.1	79.2
VGG-16 [66]	89.6	89.0	89.0	88.5	88.6	91.0
Alex-Net [12]	87.3	86.4	83.4	81.0	88.3	88.7
**VisNet**	**93.4**	**91.3**	**90.8**	**89.5**	**96.8**	**94.0**

Bold values are the highest performances in the columns.

**Table 7:** Performance comparison of multiple models on three datasets. Experimental results obtained by calculating the mean square error (MSE) of validation and test sets.

Models	FOVI Dataset (Long-Range)		FOVI Dataset (Short-Range)		FROSI Dataset (Short-Range)	
	Val. Error	Test Error	Val. Error	Test Error	Val. Error	Test Error
Simple neural network [63]	19.5	15.8	18.6	17.9	12.9	11.0
Relative SVM [29]	14.7	13.1	16.3	14.5	13.1	11.9
Relative CNN-RNN [29]	12.4	12.2	11.2	11.7	11.8	10.7
ResNet-50 [65]	11.8	10.7	14.4	13.7	9.5	9.7
VGG-16 [66]	10.1	9.9	10.4	10.0	8.0	7.8
Alex-Net [12]	11.4	10.9	11.3	11.1	9.1	8.9
**VisNet**	**9.7**	**9.5**	**9.9**	**9.8**	**7.5**	**7.5**

Bold values are the lowest error in the columns. *Val. error*—validation error.

**Table 8:** Performance of multiple models trained using original (ORG), spectral filtered (SCP), and FFT filtered (FFT) images of three datasets. The weighted sum rule (Sum) was used to combine the obtained results of each trained network (unit in %).

Models	FOVI Dataset (Long-Range)				FOVI Dataset (Short-Range)				FROSI Dataset (Short-Range)			
	ORG	SPC	FFT	Sum.	ORG	SPC	FFT	Sum.	ORG	SPC	FFT	Sum.
Simple neural network [63]	45.2	41.9	51.3	49.8	38.1	36.1	37.6	37.4	57.0	52.1	53.8	55.0
Relative SVM [29]	68.5	59.0	61.1	65.6	57.0	55.6	58.3	57.0	72.2	69.6	70.5	71.2
Relative CNN-RNN [29]	81.3	77.0	75.6	78.0	78.4	71.6	75.5	76.0	84.4	73.5	79.3	79.8
ResNet-50 [65]	70.9	61.9	73.4	71.2	68.6	60.0	67.5	64.8	79.2	76.7	78.0	78.1
VGG-16 [66]	**89.0**	79.7	81.0	86.7	**88.5**	79.8	81.1	85.2	**91.0**	86.4	88.2	89.3
Alex-Net [12]	86.4	76.0	78.5	82.1	81.0	76.3	77.1	78.2	88.7	79.0	83.6	84.7
VisNet (single STREAM)	70.1	70.5	79.1	74.7	72.7	69.4	71.9	71.3	78.8	75.6	80.3	78.8

Bold values are the highest performance in the dataset columns. *ORG*—original input images, *SPC*—spectral filtered input images, *FFT*—FFT filtered input images.

**Table 9:** Comparisons of VisNet and multiple CNNs fusion results (unit in %).

Models	FOVI Dataset (Long-Range)	FOVI Dataset (Short-Range)	FROSI Dataset (Short-Range)
VisNet	91.3	89.5	94.0
VGG-16/ORG + VisNet	**91.8**	90.0	94.15
VGG-16/SPC + VisNet	91.5	89.53	94.25
VGG-16/FFT + VisNet	91.3	89.8	**94.4**
VGG-16/ORG + VGG-16/SPC + VGG-16/FFT + VisNet	91.40	**91.8**	94.2

Bold values are the highest performance in the columns.

**Table 10:** Configurations of networks and classifications results of the five best solutions on three datasets.

Net. Name	Convolutional Layers								Class. Acc. [%]
	C-1	C-2	C-3	C-4	C-5	C-6	C-7	C-8	
CNN-1	32 * FS 3 × 3 MS 199 × 149 Stride 2	64 FS 3 × 3 MS 99 × 74 Stride 2	128 FS 3 × 3 MS 49 × 36 Stride 2	256 FS 3 × 3 MS 24 × 17 Stride 2	512 FS 3 × 3 MS 11 × 8 Stride 2	–	–	–	78.3 - 75.4 - 81.1
CNN-2	32 FS 3 × 3 MS 199 × 149 Stride 2	64 FS 3 × 3 MS 99 × 74 Stride 2	128 FS 3 × 3 MS 49 × 36 Stride 2	128 FS 1 × 1 MS 49 × 36 Stride 1	256 FS 3 × 3 MS 24 × 17 Stride 2	256 FS 1 × 1 MS 24 × 17 Stride 1	–	–	86.0 - 83.1 - 87.2
CNN-3	64 FS 1 × 1 MS 400 × 300 Stride 1	64 FS 3 × 3 MS 398 × 298 Stride 1	128 FS 1 × 1 MS 199 × 149 Stride 1	128 FS 3 × 3 MS 197 × 147 Stride 1	256 FS 1×1 MS 98×73 Stride 1	256 FS 3 × 3 MS 48 × 36 Stride 2	256 FS 1 × 1 MS 48 × 36 Stride 1	–	**91.3** **-** **89.5** **-** **94.0**
CNN-4	64 FS 1 × 1 MS 400 ×3 00 Stride 1	64 FS 3 × 3 MS 199 × 149 Stride 2	128 FS 1 × 1 MS 199 × 149 Stride 1	128 FS 3 × 3 MS 99 × 74 Stride 2	256 FS 1 × 1 MS 99 × 74 Stride 1	256 FS 3 × 3 MS 49 × 36 Stride 2	512 FS 1 × 1 MS 49 × 36 Stride 1	512 FS 3 × 3 MS 24 × 17 Stride 2	84.0 - 82.3 - 78.2
CNN-5	64 FS 3 × 3 MS 400 × 300 Stride 1	128 FS 3 × 3 MS 199 × 149 Stride 2	256 FS 1 × 1 MS 199 × 149 Stride 1	256 FS 3 × 3 MS 99 × 74 Stride 2	256 FS 1 × 1 MS 99 × 74 Stride 1	512 FS 1 × 1 MS 99 × 74 Stride 1	512 FS 3 × 3 MS 49 × 36 Stride 2	512 FS 1 × 1 MS 49 × 36 Stride 1	83.0 - 77.1 - 71.7

*Net. name*—network name; *—number of filters; *FS*—filter size; *MS*—size of feature map; *Class. Acc*.—Classification accuracy for 3 ranges: (long-range FOVI)–(short-range FOVI)–(short-range FROSI); Padding = 0 for each convolutional layer; Fully connected layers (FC1, FC2, FC3) = (1024, 2048, 4096), respectively; Number of outputs = 41, 21, 7.
